# “The genie is out of the bottle”: a qualitative study on the impact of COVID-19 on continuing professional development

**DOI:** 10.1186/s12909-024-05498-9

**Published:** 2024-06-06

**Authors:** Sophie Soklaridis, Rowen Shier, Rabia Zaheer, Michelle Scully, Betsy Williams, Sam J. Daniel, Sanjeev Sockalingam, Linda Dang, Martin Tremblay

**Affiliations:** 1 Department of Education Services, Centre for Addiction and Mental Health, Toronto, ON Canada; 2 Department of Psychiatry, Temerty Faculty of Medicine, University of Toronto, Toronto, ON Canada; 3 Department of Family and Community Medicine, Temerty Faculty of Medicine, University of Toronto, Toronto, ON Canada; 4 The Wilson Centre, University Health Network, University of Toronto, Toronto, ON Canada; 5 Professional Renewal Centre, Lawrence, KS USA; 6 Wales Behavioral Assessment, Lawrence, KS USA; 7 Department of Psychiatry, School of Medicine, University of Kansas, Lawrence, KS USA; 8 Department of Pediatric Surgery, McGill University, Montréal, Québec Canada; 9 Slaight Family Centre for Youth in Transition, Centre for Addiction and Mental Health, Toronto, ON Canada; 10 Continuing Professional Development Department, Fédération des médecins spécialistes du Québec, Montréal, Québec Canada; 11 Centre for Addiction and Mental Health, 1025 Queen Street West B1 – 2nd Floor, Room 2300, Toronto, ON M6J 1H4 Canada

**Keywords:** Continuing professional development, Medical education, COVID-19, Leadership, Learning, Innovation, Delivery modalities, Hybrid, Accessibility, Emergency preparedness

## Abstract

**Background:**

The onset of the COVID-19 pandemic catalysed a monumental shift in the field of continuing professional development (CPD). Prior to this, the majority of CPD group-learning activities were offered in-person. However, the pandemic forced the field to quickly pivot towards more novel methods of learning and teaching in view of social distancing regulations. The purpose of this study was to obtain the perspectives of CPD leaders on the impact of the pandemic to elucidate trends, innovations, and potential future directions in the field.

**Methods:**

Semi-structured interviews were conducted between April-September 2022 with 23 CPD leaders from Canada and the USA. Interviews were audio-recorded, transcribed, and de-identified. A thematic analysis approach was used to analyse the data and generate themes.

**Results:**

Participants characterised COVID-19 as compelling widespread change in the field of CPD. From the interviews, researchers generated six themes pertaining to the impact of the pandemic on CPD: (1) necessity is the mother of innovation, (2) the paradox of flexibility and accessibility, (3) we’re not going to unring the bell, (4) reimagining design and delivery, (5) creating an evaluative culture, and (6) a lifeline in times of turmoil.

**Conclusion:**

This qualitative study discusses the impact of the pandemic on the field of CPD and leaders’ vision for the future. Despite innumerable challenges, the pandemic created opportunities to reform design and delivery. Our findings indicate a necessity to maintain an innovative culture to best support learners, to improve the healthcare system, and to prepare for future emergencies.

**Supplementary Information:**

The online version contains supplementary material available at 10.1186/s12909-024-05498-9.

## Background

Continuing professional development (CPD) in the health professions comprises education activities which serve to maintain, develop, and/or increase health professionals’ competence and performance [1, 2]. Research suggests that CPD is integral to safe and effective practice, and that a lack of engagement in CPD activities results in risks to patients, staff, and organisations [3].

With the global onset of the Coronavirus disease 2019 (COVID-19) pandemic in March 2020, the field of CPD was met with unprecedented disruption, echoing the turmoil felt across society. Prior to this, formal CPD opportunities were predominantly provided in person, through conferences, courses, seminars, workshops, and grand rounds [4]. Similarly, informal CPD activities traditionally took place through person-to-person mentoring, coaching [5], or membership in a community of practice [6]. Despite an abundance of promising digital technology pre-pandemic, CPD organisations largely exhibited resistance towards embracing novel delivery methods [7]. However, with the advent of COVID-19, ushering in an era of self-isolation and physical-distancing measures [8], CPD providers were compelled to adapt their approach to delivery and accreditation [9]. The urgency of this transformation was further elevated by the centrality of CPD in equipping clinicians with credible information and the necessary skills to navigate the evolving health crisis [10]. In this qualitative study, we conducted semi-structured interviews with leaders in the field of CPD across Canada and the USA to understand the trends and innovations in teaching and learning that emerged from the pandemic and to explore how leaders imagine the future of CPD education. We discuss shifting attitudes and the emergence of new priority areas. This work contributes significantly to the literature by illuminating the field’s own standpoint on its successes and shortcomings during the pandemic and ambitions for the future. We note that at the time of writing of this article the World Health Organization has declared an end to COVID-19 as a Public Health Emergency of International Concern [11], likewise there appears to be a prevailing trend to downplay the pandemic as a past tense phenomenon in an effort to return to a semblance of normalcy [12]. However, the implications of the pandemic for CPD continue to reshape the landscape, necessitating ongoing adaptations and an enduring commitment to address the evolving needs of clinician learners.

## Methods

### Design

Given the exploratory nature of our study, a qualitative research design was chosen as the most appropriate method for eliciting the perspectives of CPD leaders.

### Participants and recruitment

To meet the inclusion criteria, participants had to be: (1) CPD leaders, (2) work in Canada or the USA, and (3) be fluent in written and verbal English. Our criteria for a “CPD leader” included involvement in CPD scholarship (discovery, integration, application, and/or teaching) [13], while holding a formal leadership position *or* having 10 + years of experience in the field.

A total of 23 participants were recruited using purposive and convenience sampling [14]. This combined approach allowed us to specifically recruit individuals with expertise in CPD leadership and the knowledge needed to answer our research question. It also provided an opportunity to ask individuals within our professional network to suggest colleagues who might be interested in participating in our study. To capture variability and diversity in perspectives, participants were selected from a range of institutions (universities, colleges, and academic health centres) and geographical regions from Canada (British Columbia, Manitoba, Nova Scotia, Ontario, Quebec) and the USA (California, Illinois, Kentucky, Maryland, Ohio, Tennessee, Wisconsin). Participants’ experience in the field ranged from 2 to 35 years, and multiple participants (*n* = 12) held dual roles as CPD professionals and medical doctors.

Prospective participants were contacted by the principal investigator (SSok) through email to inform them about the study. Interested participants were thereupon forwarded to a member of the research team (RS, RZ), who was not known to them and had no affiliation with any of the CPD offices, to recruit and initiate the consent and interview process.

### Data collection

Interviews, approximately 60 min in length, were conducted between April and September 2022 by two members of the research team (RS, RZ) via WebEx video conferencing [15]. Research Electronic Data Capture (REDCap), a secure web-based software platform, was used to obtain e-consent from participants [16, 17]. Interviews were audio-recorded, transcribed, and de-identified. Quotes included in this article convey key messages from the wider data set and have been lightly edited for clarity. Open-ended interview questions covered topics including: (1) How has COVID-19 disrupted CPD? (2) How are CPD organisations adapting to the new reality? and (3) Potential future directions in CPD? (See Additional file 1 for the complete set of interview questions).

### Data analysis

We conducted a thematic analysis using the six-step process detailed by Braun and Clarke [18]. After a familiarisation period, authors (RS, RZ, SSok) independently reviewed a subset of transcripts before collaboratively generating an initial codebook. Subsequently, all 23 transcripts were uploaded into Dedoose 9.0.54 qualitative-analysis software [19] and coded by authors LD, MS, and RS. Authors (LD, MS, RS, RZ, SSok) met routinely to discuss coding, reflexive memos, and to identify recurring patterns in the data. Next, authors (BW, LD, MS, MT, RS, RZ, SSok) organised codes into preliminary themes, ensuring that all codes captured under one theme were interrelated. Iterative re-coding and analysis were performed to refine themes until they were reflective of the complete data set. This article presents a subset of the data gathered during the study. A portion of the data was separately published in consideration of its distinctive thematic elements warranting detailed examination [20].

### Trustworthiness

In this study we employed intercoder reliability [21], negative case analysis [22], and reflexivity [23] to enhance the trustworthiness of our analysis. Double-coding was implemented for the first three transcripts until coder consensus was established. Throughout coding and analysis, authors (LD, MS, RS, RZ) engaged in reflexive memoing to extract meaning from the data, promote critical thinking, and enhance team dialogue [24]. Furthermore, given our diverse personal and professional identities, we frequently engaged in discussion to determine appropriate research paradigms for our research objectives and to investigate our collective subjectivity [25].

### Ethics

Research Ethics Board approval was obtained from the Centre for Addiction and Mental Health (REB 023/2021).

## Results

From the data, we generated six themes related to the impact of the pandemic on CPD organisations: (1) necessity is the mother of innovation, (2) the paradox of flexibility and accessibility, (3) we’re not going to unring the bell, (4) reimagining design and delivery, (5) creating an evaluative culture, and (6) a lifeline in times of turmoil (See Table 1).

**Table 1:** Summary of themes and exemplar quotes

Theme	Exemplar Quotes
1. Necessity is the mother of innovation	“I guess if you say, necessity is the mother of invention, we innovated. But it’s not so much innovation as it was in some cases, we were just *forced* to use the tools that were there in order to connect and interact.” – P013
2. The paradox of flexibility and accessibility	“I think added flexibility for people is something that’s really important. A “7 a.m. Grand Rounds” where half of your female faculty couldn’t attend because they were dropping children off at school, but now they’re online and can actually participate. But I also think it really needs to be tempered because just because you can put something online and ask that a clinician do it in their spare time, it just erodes into the time that they’re not supposed to be doing work.” – P003
3. We’re not going to unring the bell	“I foresee us living in a hybrid environment for a long time and having that extra flexibility for people to determine how they want to participate.” – P020
4. Reimagining design and delivery	“I think CPD going forward is no longer the kind of didactic, bums in seats […] I think CPD needs to be workplace-based, practice-based, able to do where you are at, when you have time to do it, with the space and tools that you have.” – P009
5. Creating an evaluative culture	“As leaders in CPD, we need to create a culture of finding new ways. We need to test what we’re actually doing and gather data and show whether or not it’s working.” – P013
6. A lifeline in times of turmoil	“I think we need to think about how CPD can help institutions prepare for the next pandemic. We’re going to have another pandemic at some point, how do we learn from this so that we’re better prepared to help our institutions, and how can we be strategic players in that so we can ramp up much more efficiently and less chaotically the next time we have to deal with an emergency like this.” – P014

### Theme 1: necessity is the mother of innovation

According to our participants, prior to the pandemic, there was widespread reluctance within CPD to deviate from ‘traditional’ ways of delivery, namely in-person education. The field showed little inclination towards the integration of emerging technologies or alternative educational approaches, holding firm in a ‘don’t fix what isn’t broken’ mentality.

“Healthcare providers are initially very resistant to new technology; we don’t want to play with new technology. We only want ‘safe’ stuff.” – P015.

The disruptions of the pandemic engendered a need for adaptation within CPD organisations. Many participants commented that change was long overdue, specifying that the required tools and technologies already existed, however, implementation had been limited. Against this setting, the pandemic brought forth an opportunity to leverage these preexisting innovations.

“Technology was already disrupting but people had a choice […] COVID took those choices away. It accelerated the use of technologies that already existed which people were struggling to get deployed.” – P008.

“I guess if you say, necessity is the mother of invention, we innovated. But it’s not so much innovation as it was in some cases, we were just *forced* to use the tools that were there in order to connect and interact.” – P013.

Participants noted that the pandemic fundamentally shifted decision-making mechanisms within their organisations. For CPD course developers this comprised the implementation of rapid knowledge dissemination strategies and swift determinations regarding modalities and formats. Similarly, CPD accreditors created rapid response teams to expedite their procedures. Thus, previously arduous hierarchical processes were replaced with rapid and targeted decision-making approaches.

“We didn’t have the luxury of saying, okay, we can work three months with a planning committee. Everything we did in terms of identifying subject matter experts, how we engage them, the frequency we engage them, all of our processes that were tried and true for so many years, we just had to stop and re-design given this environment. I think that’s innovation.” – P002.

However, as illustrated by one participant, as the health crisis has waned, some of the conventional hierarchical practices in CPD have begun to resurface:

“In the beginning I think there was more space for innovation because a lot of the hierarchy that goes along with decision making within any institution was taken away because people realised that you had to do things, and you had to do things quickly. Some of that hierarchy has come back now.” – P007.

Nevertheless, participants determined that the pandemic imposed a much-needed shift in prevailing attitudes towards innovation:

“In the past when you brought those [technologies] up, people’s eyes glazed over […] Now they’re like, oh really, I could do that? I can operate an anaesthesia machine remotely? Tell me more! Before it was, you’ve got to be kidding me, that’s dangerous.” – P008.

### Theme 2: the paradox of flexibility and accessibility

The response to virtual learning was mixed. CPD providers faced many challenges as they navigated unfamiliar technology, striving to make the content both meaningful and engaging. Additionally, concerns emerged about the potential loss of ‘hands-on’ experience, prompting questions about pedagogical quality and transferability:

“Some of it we have noticed from the residents that have been coming through, and certainly from the course that I developed, I think people are missing out on some of the hands-on training that virtual CPD cannot provide.” – P023.

Despite pedagogical uncertainties, there appeared to be a marked rise in CPD programming attendance, with many attributing this to increased accessibility and flexibility. The pivot towards virtual CPD offerings enabled participation from a broader community of clinicians in ways that were not possible pre-pandemic. Notably, some participants described how virtual platforms enhanced accessibility.

“The good thing has been the reach. Many of the providers are saying that they’ve been able to reach individual learners and audiences, including those internationally and in more rural areas that we were not able to reach before.” – P002.

“For example, in India, women emergency physicians are never resourced to go to international conferences. The guys go, and the women stay behind and man the fort. Having something remote can provide access to education.” – P016.

“For our young membership it’s good because they can attend even if they have a baby at home.” – P012.

Furthermore, one participant highlighted the utility of virtual CPD for learners with diverse learning needs:

“There are people that have hearing disabilities that really appreciate being online, as it’s a controlled environment […] They can really focus and concentrate on what is being said, and with the addition of subtitles, it’s really reducing some accessibility challenges.” – P019.

In contrast, some participants were less optimistic about virtual education. Although women were cited as potential benefactors of virtual CPD, they were also asymmetrically burdened by household and parental obligations during the pandemic, thus limiting their capacity to meaningfully engage in CPD.

“Women’s work was impacted significantly for clinicians and non-clinicians, because even though we talk about equality and we’re working outside our homes, we still do far more at home. I could give you examples of two or three PhD researchers in CPD who I had meetings with, and their partner got the good computer, the office, the quiet space in the basement, and they were in the TV room with SpongeBob playing […] I suspect when we look at the literature we’ll discover that a lot of guys wrote papers during COVID, and a lot of women just didn’t have the bandwidth.” – P016.

Moreover, participants signalled the challenges that older clinicians experienced with the rapid introduction of new technologies:

“A lot of our senior members closed their practices, and retired either because of the lack of patients, or the lack of their ability to acquire the technology that they needed to see their patients virtually and confidently. It really put such a burden on them.” – P023.

Similarly, several participants cautioned against adding superfluous virtual education to a busy clinician’s life. Given the inordinate rate of burnout associated with the pandemic, participants felt that an overabundance of programming compromised both clinicians’ task attention and their work/life boundaries.

“I think added flexibility for people is something that’s really important. A “7 a.m. Grand Rounds” where half of your female faculty couldn’t attend because they were dropping children off at school, but now they’re online and can actually participate. But I also think it really needs to be tempered because just because you can put something online and ask that a clinician do it in their spare time, it just erodes into the time that they’re not supposed to be doing work.” – P003.

“Normally [clinicians] would book off their practice and would go to a conference for two days. That was no longer possible. So, moving online, even though there was greater flexibility, there was still that sense that I might have to stop at any moment and take a call.” – P009.

### Theme 3: we’re not going to unring the bell

As pandemic restrictions eased, many organisations moved towards a “hybrid,” “blended,” or “hyflex” model of delivery.^1^ Despite the lack of clarity around these terms, there was widespread expectation that this model will remain prominent.

“I foresee us living in a hybrid environment for a long time and having that extra flexibility for people to determine how they want to participate.” – P020.

“The definitions are constantly changing because we haven’t really defined what it’s going to look like after the pandemic. There are all of these words that we’re using like hyflex, hybrid, but there’s no concrete definition. And there are ways of collaborative learning with technology that haven’t even been defined yet.” – P017.

Conversely, several participants acknowledged that virtual delivery may *not* always be preferred. While some learners appreciated this new mode of delivery, many lamented the loss of networking and social connection, as well as the Zoom fatigue associated with videoconferencing. Participants substantiated this through examples such as clinicians’ eagerness to resume in-person events despite the presence of tangible barriers, including travel time:

“We offered [a conference] virtually and in-person. The virtual numbers were so low that we cancelled the virtual. We have about 260 mostly rural doctors coming down to a location in the province because networking and getting out of the communities was so important.” – P010.

“The number one complaint is we can’t break bread. We can’t talk with one another. We can’t meet our future employers. Maybe they want to speak with somebody on the east coast or the west coast. How are the programs different? We can have these conversations, but it’s limited.” – P017.

Additional considerations pertaining to hybrid events can act as deterrents. For instance, these events can be more cost prohibitive, requiring additional staffing and information technology infrastructure. This can make hybrid delivery especially challenging to implement in smaller scale contexts.

“Many [organisations] were doing hybrid [events]. We tried to do that, but we had such low numbers that it wasn’t worth the cost. It costs so much more to do both.” – P010.

“The biggest problem we have right now is the smaller teams cannot do hybrid CPD. We tried to help them, but it was not a success […] They are now starting to do face-to-face CPD, and they did virtual CPD during the pandemic, but the hybrid one is really, really difficult to implement.” – P004.

Hybrid events were also described as more challenging to host than *exclusively* virtual or in-person events. During the initial transition to virtual delivery, both learners and educators had to navigate unfamiliar technology. Educators had to adapt their content and find ways to meaningfully engage the audience in the absence of body language and the inclination of learners to multitask. These challenges are compounded in a hybrid environment where educators must decipher how to effectively engage both audiences simultaneously.

“But if we do the two together, that’s where I think the challenge is going to be. How do you integrate both an in-person and a virtual component, and be able to satisfy both audiences, and have everyone feel included in a meaningful way?” – P010.

Despite these concerns, most participants concurred that the field has changed forever:

“What it’s going to look like after the pandemic is really hard to say because everything is changing so quickly right now, but we’re not going to unring the bell. You’re not going to get to a point where we’re going to go back to the way things were before the pandemic as far as teaching and CPD goes. We’re on a very steep learning curve right now.” – P017.

### Theme 4: reimagining design and delivery

In addition to the longevity of hybrid education, participants shared how CPD could further refine delivery methods to maximise learning and drive practice change. They commented on anticipated learning model trends which included: micro and practice-based learning; learner-centric and personalised learning; workplace-based and in situ learning; collaborative learning (team-based and interprofessional education); longitudinal learning; and mentoring/coaching. Further research is needed to determine optimal learning environments, approaches, and design.

“I think CPD going forward is no longer the kind of didactic, bums in seats […] I think CPD needs to be workplace-based, practice-based, able to do where you are at, when you have time to do it, with the space and tools that you have.” – P009.

“The future of CPD really has to be flexible, individualised, point in time, work integrated, leveraging people’s experiences. That sounds almost impossible today. How do you do that? Other than having a one-to-one educator learner structure. That’s my immediate thought. But that’s because we haven’t figured it out yet.” – P005.

Participants similarly contemplated the use of technology to enhance CPD education, including social media, electronic medical records, virtual reality, and artificial intelligence (AI).

“I have a feeling long-term-wise there are a lot of activities that AI could replace. For example, instead of speaking to a coach about a problem, you could get that basic interaction with AI, and you could go back to it as often as you needed as opposed to using those human resources, which is often where we run out of steam. I see [AI] as a potential significant addition that would make the delivery of CPD easier, but still effective and impactful.” – P018.

In tandem with reimagined tools and methods for education, a few participants underscored the importance of involving key stakeholders in education design, emphasising the importance of these voices in the conception, implementation, and delivery of CPD.

“Scholarship in innovation without ensuring that the end user is at the table with the implementation in the design of what you’re trying to create, I think it is potentially an oversight. Because the best designed, best intended education, with the most robust outcomes, if it cannot be adopted and integrated into the workflow of your target audience, it’s not going to matter.” – P003.

“I think one way to improve things would be to have a patient, somebody who is using the health services, to have them in our meetings, and to have them take a look at the evaluation that we do of our program.” – P004.

### Theme 5: creating an evaluative culture

Participants called for improved evaluation strategies to assess the quality of CPD education as well as learner and, ultimately, patient outcomes. Historically, evaluation has been insufficient and under-resourced, however, post-COVID, there appears to be increasing momentum towards a data-driven approach to improving service delivery.

“As leaders in CPD, we need to create a culture of finding new ways. We need to test what we’re actually doing and gather data and show whether or not it’s working.” – P013.

“Is the quality of what is produced virtually of higher quality, lesser quality, or the same quality as in-person CPD? – P023.

“We need to have some evaluations that really do reflect likelihood to change. It no longer cuts the mustard to say we served ‘x’ number of people, and they liked it.” – P011.

Furthermore, evaluations should be multipronged, longitudinal, and demonstrate tangible outcomes:

“All education should be designed around outcomes; improved patient outcomes, improved environment for people to practise in, increased value, decreased cost; there are a million.” – P003.

However, some participants noted that barriers such as limited resources, including time and money, continue to hinder meaningful evaluation initiatives.

“I think that resources don’t exist to measure outcomes and design activities in a meaningful way. Pushing on [CPD] offices to create education that leads to meaningful outcomes, again without providing the resources to measure the patient outcomes or collect and analyse the data is unfair.” – P003.

Evaluation is integral to demonstrating the value of CPD. Despite the increasing recognition of its importance, much work remains to ensure it is ingrained within the field.

“If we can’t show our value to decreasing costs and improving patient outcomes in the long-term, I do believe there is a significant threat to CPD education.” – P002.

“Evaluation culture is starting, but we still have so much teaching to do to make sure that it’s something that is well accepted.” – P004.

### Theme 6: a lifeline in times of turmoil

Finally, participants remarked that the value of CPD became heightened during the pandemic among medical professionals and the public. Amid chaos and confusion, it became a trusted source of information providing life-saving education about the virus, ultimately, becoming a lifeline for those working in medicine.

“It has impressed me how much CPD can do to be of value and a change in what is happening to the population. CPD plays a critical role in not only just delivering education […] We are a trusted source.” – P010.

“I think the value of CPD has been significantly elevated. It became a very important, valued, sought after solution to a complex problem. And so, it has elevated its position in health professions.” – P005.

Moreover, CPD attained an indispensable status in the reorganisation of the healthcare system, expanding their mandate and facilitating interprofessional and cross-organisational collaboration to manage the crisis.

“CPD became a forum to manage this health crisis. This has shown the power of education and bringing the leaders together in helping to manage a health crisis or anything healthcare related.” – P010.

“We helped our colleagues organise themselves. We reorganised the healthcare system. The nurses were gone, so what do you do in the clinics now? How can we help them? So CPD now includes management. That is something that we didn’t do before. We needed to do CPD for team leaders. They needed that. They needed the help to manage their team. All the organisation of the clinic, of the hospital, how can we be there for them, and teach them how to do it? The subjects of CPD exploded with the pandemic.” – P004.

To best prepare for the next emergency, participants extracted key lessons from COVID-19, which included adaptability, rapid decision-making, and collaboration.

“Today it’s COVID. Tomorrow it may be something else. So, educators need to find that adaptability.” – P005.

“I think we need to think about how CPD can help institutions prepare for the next pandemic. We’re going to have another pandemic at some point, how do we learn from this so that we’re better prepared to help our institutions, and how can we be strategic players in that so we can ramp up much more efficiently and less chaotically the next time we have to deal with an emergency like this.” – P014.

However, some participants expressed concern that the lessons from this pandemic may lose traction. Notably, one participant positioned this apprehension in the context of the 2002–2004 severe acute respiratory syndrome (SARS) outbreak, highlighting that much of the knowledge gained from that crisis was overlooked. This led to inadequate emergency preparedness resulting in the reactionary approach to innovation during COVID-19.

“[An author] wrote an article about SARS1 in which he outlined all of the things that CPD should become because of SARS1. It included using technology more, more longitudinal, more networking. SARS1 was quite short lived, although it was still very impactful. But there we were ten years later reading the article and it told us exactly what we should have been doing, and we would have been very prepared […] Most people will probably say that CPD has changed forever. I think it’s changed given that we’ve been in this pandemic for quite a long time. I’m still not convinced CPD has changed forever. I don’t know if we’ve changed our mindset enough as a group of professionals. I think we’re still thinking about knowledge transfer in a very traditional way. I probably sit on the side of, we will go back to as it was, more than we will hold on to the changes.” – P013.

Irrespective of participants’ viewpoints on the lasting impact of COVID-19 on CPD, there was widespread agreement that a comprehensive examination of both the failures and successes of the field’s response to the crisis is essential.

“Let’s say we get another pandemic, how can CPD be more effective in rolling out information? It’s hard to teach when information is not reliable and is ever-changing. How can CPD offices in the future, using COVID as an example, find out what went wrong, what went right?” – P011.

“By the time something like a crisis happens it’s too late. It’s too late to innovate. You just respond.” – P013.

## Discussion

To our knowledge, this is the first study exploring the perspectives of CPD leaders regarding the impact of COVID-19 on CPD and potential directions post-pandemic. We found that the pandemic incited a long-awaited transformation in teaching and delivery, although some CPD leaders expressed reservations about the endurance of these changes. Historical evidence attests that periods of turmoil act as powerful catalysts for innovation. Our participants substantiated that the pandemic acted as an inflection point for CPD. McMahon [10] highlights that the urgent demand for new solutions prompted CPD organisations to adopt a more decentralised approach to decision-making, thus fostering an environment ripe for innovation. In alignment with the broader literature, our findings indicate that the most prominent change experienced within CPD was the transition to virtual education which was associated with a significant surge in CPD attendance at the beginning of the pandemic [10, 29–31].

Increased flexibility and accessibility have been touted as the primary advantages of online learning [29, 32]; however, there exists a duality of sentiments regarding the implications for historically excluded groups. Some scholars contend that the flexibility offered by virtual delivery has the potential to eliminate systemic barriers for equity-denied groups [29, 32]. In particular, COVID-19 may have increased access to medical education content for women [29], parents of young children [33], individuals from remote communities [29, 34], and international audiences [35] who may face barriers to in-person attendance. Conversely, we found that in certain instances, virtual delivery impeded clinicians’ ability to meaningfully engage in CPD, particularly for women and senior clinicians. This duality is recognized in the literature which cautions that virtual CPD may inadvertently reinforce gender disparities. There is evidence that women were disproportionately burdened by household and childcare duties during the pandemic, thereby potentially reducing their capacity to engage in virtual CPD [33, 36]. While additional research on this topic is warranted, in light of these nascent findings, CPD program developers should consider the divergent experiences that exist among different populations when assessing content delivery modalities.

Despite the challenges listed above, our findings suggest a prevailing interest in retaining the virtual component of medical education which may significantly alter the nature of CPD delivery moving forward. In fact, some scholars argue that learning preferences have been permanently altered by the pandemic [31]. Given undergraduate and graduate medical education students also pivoted to virtual learning, with research indicating a high degree of acceptance [37], this may further reinforce the enduring nature of the virtual component. While the wider literature indicates a preference towards “hybrid” education [31, 32], there remains gaps in knowledge regarding this modality. Our research indicates a foremost need to obtain definitional clarity regarding what constitutes “hybrid,” “hyflex,” and/or “blended” education. In addition, there remains a paucity of literature on the unique considerations associated with meaningful engagement during hybrid events. Preliminary research has begun to contribute to this gap, for example, Gottlieb et al. [35] found that using multiple mediums can introduce challenges with synchrony that dilute the quality of experience for both in-person and virtual audiences. The authors suggest that enlisting technology specialists, creating opportunities for interactivity and engagement, and ensuring that content is appropriate for digital format are crucial to enhancing hybrid events. In proceeding forward, the creation of best practices in hybrid CPD is imperative.

In conjunction with this pivot towards virtual formats, our participants asserted that greater change is needed in CPD to sustain relevance in the face of evolving societal circumstances. These calls to reform CPD predate the pandemic [38]. Our participants exhorted the investigation and adoption of innovative learning models such as team-based, workplace, and practice-based learning, which gained momentum during the pandemic. These approaches are crucial for thinking and moving beyond professional silos [39], enhancing knowledge translation through practice [40], and ensuring learned skills and knowledge are relevant to patient care [41], respectively. Moreover, several of our participants expressed a keen interest in expanding the use of innovative technology which they experimented with during COVID-19, albeit with varying degrees of success. This included the use of trending digital tools such as social media [42], podcasting [43], and AI [44], which have been explored by other scholars.

Our participants further voiced the need for CPD to be informed by systematic evaluation to measure learning and clinical outcomes. Scholars have long advocated for robust evaluation and assessment practices in CPD, contending that the development of effective learning interventions requires evidence-based content with well-defined, attainable, and measurable learning outcomes [45]. This can help bolster the value of CPD by providing objective indicators that demonstrate how CPD activities improve clinician performance, augment healthcare quality, and improve cost effectiveness [46]. While outcome evaluation is crucial, a recent scoping review [47] examining CPD evaluation techniques revealed major gaps; the authors argue for comprehensive approaches that integrate process evaluation and that are guided by theoretical frameworks. We contend that this endeavour is not possible without meaningful support from CPD offices, through both increased financial and human resources.

As a final consideration, our participants underscored the need for CPD to adopt a proactive stance in preparation for future crises. However, in light of CPD’s failure to implement lessons from previous emergencies, participants expressed concerns that takeaways from COVID-19 may not be internalised and applied moving forward. For example, in their article on CPD delivery after the 2003 Toronto SARS outbreak, Davis et al. [48] urged providers to develop a “flexible” emergency preparedness plan outlining the role of technology in achieving those aims. Moreover, the authors stressed the importance of transitioning CPD from “a passive, reactive model toward a multimodal, proactive, and systemic vehicle” to facilitate the dissemination of up-to-date information. These recommendations resonated with our participants, who felt that had these been effectuated, the field would have been better prepared for COVID-19. Drawing on lessons from the past with an eye to the future, three paramount COVID-19 takeaways were proposed: (1) the importance of fostering a culture of innovation, (2) promoting interprofessional and patient collaborations, and (3) demonstrating the value proposition of CPD as a lifeline for clinicians.

### Limitations

We note that because the study sample was restricted to participants from Canada and the USA, the findings should be interpreted within this specific contextual milieu and may not necessarily be transferable to other settings. While we made efforts to recruit a diverse sample (including professional backgrounds and years of experience), we did not formally collect demographic data. Finally, given this research captured perspectives during a determinate timeframe, we acknowledge that attitudes, perspectives, and experiences may have evolved as the COVID-19 situation has progressed.

## Conclusion

The pandemic presented profound implications for CPD, compelling a culture of adaptability and innovation. The challenges generated opportunities for the field to reimagine CPD design and delivery to reflect evolving societal conditions and preferences. Moreover, this allowed the field to build resilience and demonstrate its value to healthcare and greater society. We caution that while the public health emergency appears to have subsided, our research underscores the need to sustain progress made during the crisis to ensure a better “new normal.” As we move forward, we call for the CPD community to leverage this momentum and internalise lessons learned to avoid perpetuating past mistakes during inevitable future crises.

### Electronic supplementary material

Below is the link to the electronic supplementary material.

Supplementary Material 1

## Data Availability

The datasets used and analysed during the current study are available from the corresponding author on reasonable request.

## Footnotes

1 It is important to note that terms such as “hybrid,” “blended,” and “hyflex” remain ambiguous with limited understanding of their precise definitions [26]. Our participants further noted there is a prevailing trend to use the above terms interchangeably in the field of CPD. As such, for the purposes of this article, we can only offer a loose definition of “hybrid,” “blended,” and “hyflex” learning as incorporating both in-person and online components [26–28]. Further research is needed to establish comprehensive definitions in the CPD context.

## Declarations

### Authors’ information

The first (SSok), second (RS), third (RZ), fourth (MS), sixth (SJD), seventh (SS), eighth (LD), and ninth (MT) are Canadian, while the fifth (BW) is American. SSok, RS, RZ, and MS are qualitative critical social science researchers. BW, SJD, SS, and MT conduct clinical outcomes research using both quantitative and qualitative methodologies. At the time of the study, LD was a student. Five authors (SSok, BW, SJD, SS, and MT) are actively engaged in North American continuing professional development initiatives, whereas four authors (RS, RZ, MS, and LD) maintain a peripheral role in the field through research contributions. Three of the authors (BW, SJD, and SS) provide clinical care.

## Abbreviations

CPD: Continuing professional development
COVID-19: Coronavirus disease 2019
AI: Artificial intelligence
SARS: Severe acute respiratory syndrome
