# Supplementary material for: “The genie is out of the bottle”: a qualitative study on the impact of COVID-19 on continuing professional development
Source: BMC Med Educ. 2024 Jun 6;24:631. doi: 10.1186/s12909-024-05498-9 (PMC11155036; doi:10.1186/s12909-024-05498-9)
Supplement: Supplementary file 1 — Supplementary Material 1 [file 12909_2024_5498_MOESM1_ESM.pdf]

Semi-Structured Interview Guide

N.B: The interview guide represents the intended topic areas/questions. Qualitative interviews are organic, and questions may evolve/alter depending on the progression of the interview.

Topic	Questions	Probes
Icebreaker	Please tell me a little bit about your current role?	• Years of experience in CPD?<br>• What is your disciplinary background?<br>• CPD role and engagement?<br>• Can you briefly describe some scholarly activities that you are engaged in (teaching, research, QI)?
COVID-19: A disruptive moment for CPD?	What impact has the COVID-19 pandemic had on CPD in your organization?	• Did your organization shift towards a virtual teaching environment as a result of Covid? What was that shift like?<br>• Did your organization make use of digital learning tools or digital platforms? (Which ones)? (Were there any challenges)? (Is your organization continuing to use these?)<br>• During the pandemic, did your organization adopt any virtual simulation training (e.g. for emergency training or procedural skills, or standard operating procedures?)<br>• From your perspective, how did Covid affect CPD program engagement/participation? (Barriers/facilitators)?<br>• How did the pandemic impact faculty engagement/confidence?
	What was your experience like in adapting to these changes?	• What kind of challenges did you face in adapting to these changes?<br>• How did you or your organization resolve these challenges?<br>• Are there any ongoing barriers that prevent these innovations?

		• What helped you in adapting to these changes (i.e. facilitators)?
	Has the role for innovating in CPD taken on new urgency or meaning for you during this rapidly changing time? To what extent?	• What changes need to happen to adapt to these changes in teaching and learning?<br>• Is there a need for a multi-faceted approach to CPD including formal and informal, especially in the role of e-learning and distance learning?<br>• How can we innovate as the pace of change due to COVID-19 accelerates?
	Which of these changes do you think will remain after the pandemic?<br><br>What new skills are health professional educators and clinicians required to acquire for delivering CPD?	• How will the learning and working conditions prepare faculty for these readjustments?<br>• Can you share some of the “new best practices” that your organization has implemented because of COVID-19?
	What is the single most important trend/innovation in CPD research/scholarship that you think those in the field need to know about?	• Do we have clear plans on how to meet or exceed the needs of educators or researchers in CPD scholarship?<br>• Are there gaps in our understanding in the delivery or effectiveness of CPD that have emerged because of this pandemic?
Rigor in Pedagogy	Do you think COVID-19 has amplified the need for CPD scholarship to be more rigorous?	• How has CPD research evolved in the pandemic?<br>• How has the CPD pedagogy changed during the pandemic?
Organizational support for CPD	Since the COVID-19 pandemic, to what extent has your current institution supported continuing professional development?	• Funding of CPD innovations/technology<br>• Shifts in resources towards CPD
Future of CPD	What do you think the future of CPD research/scholarship is in the short (1-4 yrs) and long term (5+ years)?	• How will CPD educators be teaching clinicians in the future?<br>• Are there any differences in the future of CPD based on heath professions or the area of healthcare?<br>• What do we need to understand better in CPD because of the

	COVID-19 adaptations/changes?<br>• Is there anything else that we should know?
